# Event and Comment

**Published:** 1915-12

**Authors:** 


### DENTAL REGISTER

Vol. LXIX DECEMBER, 1915 No. 12

### EVENT AND COMMENT

WHAT HAVE WE DONE? The injunction of St. Paul that we should forget the things that are past and press forward to things to come, in some cases may not be the best thing to do. It is a good thing occasionally to take account of our situation, that in street parlance, "we may know where we are at." If we have done well it encourages us to start the next lap with more enthusiasm. Naturally we look backward at this time to see what has been accomplished during the past year. It is of course impracticable for us to review the entire course, and besides the detailed enumeration would be somewhat tiresome. While after all it is the thousands of little things and our individual relations to these events in our own lives that constitute the real sum total of development in the profession, we can not count these for we do not know them all. It is the larger events that perhaps can not be associated altogether with the individual that are at least symptomatic, if we can use such a simile, of the onward progress of the profession. There have come to us during the year a few notable events that are worth recording and that certainly mark our growth as a profession. The interest in the public propaganda for better care of the teeth has been kept up, and seems to have crystallized into more permanent and substantial methods of accomplishment. This work is being taken over by public health boards and institutions in a way that seems to promise permanence and effectiveness. The realization of the great philanthropy made possible by the Forsythe Institute in Boston, and the promise of another similar institution in the proposed Eastman Dispensary in Rochester are perhaps the most notable events along this line in the history of our profession, and they certainly indicate in no uncertain manner the fact that the general public has begun to believe what we have for so long known, that the good health of the teeth is a tremendous power for good general health. In the educational sphere we can see distinct evidences of growth. There are indications of a better appreciation of our literature, and a goodly number of valuable books have been published during the year, and the contributions to the journals and through the dental societies have been of an unusually high type. The educational institutions have increased numbers in attendance, and new buildings and improved equipments are constantly being added. One of the most notable additions to these forces is the new Evans Institute building, which gives the University of Pennsylvania what is unquestionably the finest dental educational plant in the world. One of its greatest advantages to the profession will result from the facilities this Institution now has for carrying on scientific research. We are to be congratulated that it is an important part of their plans to secure men capable of doing such work and give them every facility for doing dental research. In this connection we have just learned that the Research Commission of the National Dental Association has purchased a property valued at $50,000 in Cleveland as a central home for its research work, which is now being done in several of our university schools. What this move means or what the outcome is to be we do not now know. Several important problems are now under investigation and reports made to the National Association this summer show interesting progress, but definite conclusions are as yet unannounced. Probably the most interesting event of the year was the meeting of the Panama-Pacific Dental Congress, at San Francisco. While this was expected to be a World Congress, owing to war in Europe the attendance from abroad was comparatively small. There were about 2,000 dentists present. The work done by the Congress was after all very satisfactory, but it can not be fully valued until the proceedings have been put into print and are available for closer study.

The reorganization of various state societies has gone on steadily and by affiliating with the National Society the membership of that organization is now approximately 15,000, which is less than forty per cent. of the dentists practicing in the country. It is the hope this year that enough states will affiliate and increase their memberships to bring the total membership up to 20,000, which seems a reasonable attainment. The expenses of reorganization and the publication of the National Dental Journal has cost so much that there remains only a small balance in the treasury, not enough to print the transactions of the Congress. It is evident that if the publication of the Journal is continued or its field materially extended the dues from affiliated societies will have to be increased. This, however, was expected, and if these dues can be increased by one dollar there will be ample funds for publishing the journal as a monthly. One of the most important events of the year is the decision of the Dental Colleges to extend the time required for the dental degree to four sessions of thirty-two weeks each, beginning Oct., 1917. This will involve a considerable readjustment in the curriculum and faculties which will have to be done during the coming year. The content of the curriculum is now one of the most discussed subjects by all college men.

During the year the profession has lost several of its most distinguished men. Dr. James Truman, the great teacher and writer; Dr. Charles R. Butler, distinguished as an inventor, writer and professional organizer; Dr. Louis Jack, as a writer and practitioner; Dr. W. X. Sudduth, as scientist and author; Dr. F. D. Weisse, as teacher and author; Dr. J. B. Wilmot, as teacher and dean of the Canadian profession, and Dr. G. V. Black, the eminent scientist, teacher and author of world-wide reputation. All these men rendered great service to their beloved profession and we should honor their memories. On Wednesday Dec. 8th, at Columbus, Ohio was dedicated the statue in memory of Professor W. D. Miller. This statue has cost about $5,000 and is placed on the campus of Ohio University in Columbus which is only thirty miles from his birthplace and the place of his burial. The money has been contributed by dentists from all over the country as a tribute expressing their gratitude for the great scientific work done by Miller for the profession.

In the technical field there has been the usual interest in various departments. No significant inventions have appeared. Some improvements and refinements in instruments and materials could be mentioned, but none of marked importance have appeared. Some closer study and improvement in the various cements has kept this subject prominently under discussion during the year, but no material improvement in materials has so far resulted. On the whole we can say it has been a year of successful progress in practically every department of the profession.

